# A Novel Surgical Technique for Resuspension of Digital Pulp Tissue after Degloving Injuries

**DOI:** 10.1097/GOX.0000000000002600

**Published:** 2019-12-26

**Authors:** Michael B. Gehring, Kai J. Yang, Jenna G. Cusic, Aishu Ramamurthi, Hani S. Matloub, Anne E. Argenta

**Affiliations:** From the Department of Plastic Surgery, The Medical College of Wisconsin, Milwaukee, Wis.

## Abstract

Adherence of pulp tissue to the underlying distal phalanx is required for fundamental actions including grip, proprioception, and fine motor skills. Disruption of the fibrous septa causes sliding between the distal phalanx and overlying soft tissues, hindering basic hand function. The authors present a novel surgical technique in which the fibrous pulp septa are resuspended to the distal phalanx with bone anchors and sclerosing agents after a closed degloving injury.

Digital pulp is essential for carrying out daily activities, especially those requiring delicate dexterity. Pulp is composed of thick glabrous skin and vascularized fibrofatty tissue. Strong fibrous septa connect the skin to the underlying periosteum of the distal phalanx. This adherence allows the pulp to play fundamental roles in grip, proprioception, and sensation.^1^ Disruption of the fibrous septa with degloving-type injuries can lead to sliding of the overlying soft tissue over the bony structures and hinder normal functions. It can also lead to seroma, hematoma, and necrosis of the overlying soft tissue due to disruption of the vascular perforators.^2^ The authors describe a case report of an internal degloving injury successfully treated in a novel fashion with suture anchors and sclerosing agents.

## CASE REPORT

**Fig. 1.**
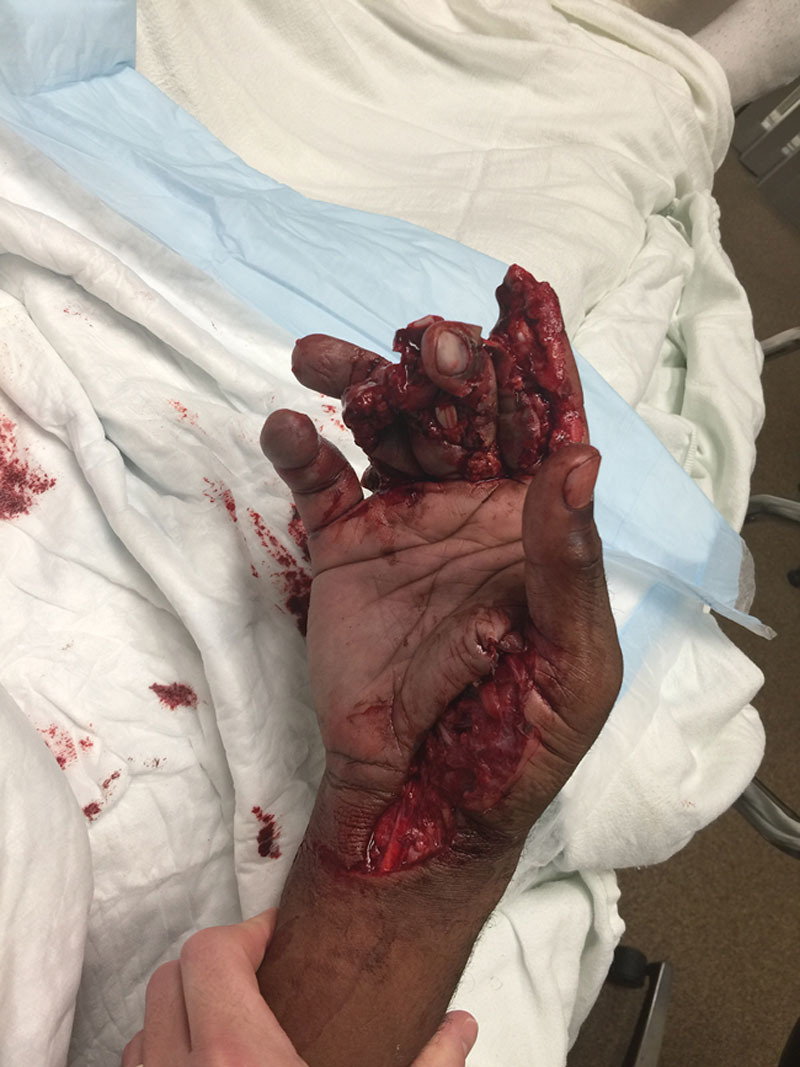
Original injury.

**Fig. 2.**
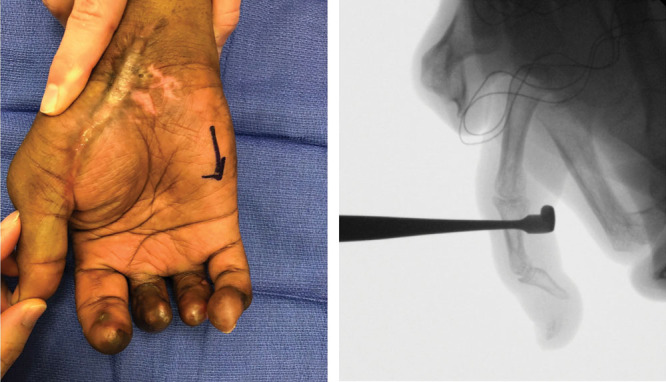
Preoperative appearance at the time of resuspension. A, Hand appearance 5 months after the original injury. B, Intraoperative x-ray demonstrating mobile volar pulp tissue.

A 39-year-old African American right-hand-dominant machinist presented with a severe right hand crush/avulsion injury involving all digits (Fig. 1). At initial inspection, the small finger was noted to have a closed internal degloving injury with intact perfusion and sensation, and a closed distal tuft fracture. The pulp tissue was able to be manipulated and passively rotated 100 degrees radially and 100 degrees ulnarly around the axis of distal phalanx. There was mild soft tissue bruising and no hematoma. The small finger was initially managed nonoperatively. Over the ensuing 5 months, the pulp tissue did not auto-adhere back to bone. Additionally, the patient developed a fixed 45-degree distal interphalangeal (DIP) joint flexion contracture with a prominent, palpable distal phalanx (P3) tip under hypermobile volar pulp tissue (Fig. 2). He complained of pain and instability when the loose pulp tissue sheered across the flexed distal P3 tip with pressure or activity, particularly grip. He denied DIP joint pain. He elected to undergo resuspension pulp tissue to the distal phalanx.

**Fig. 3.**
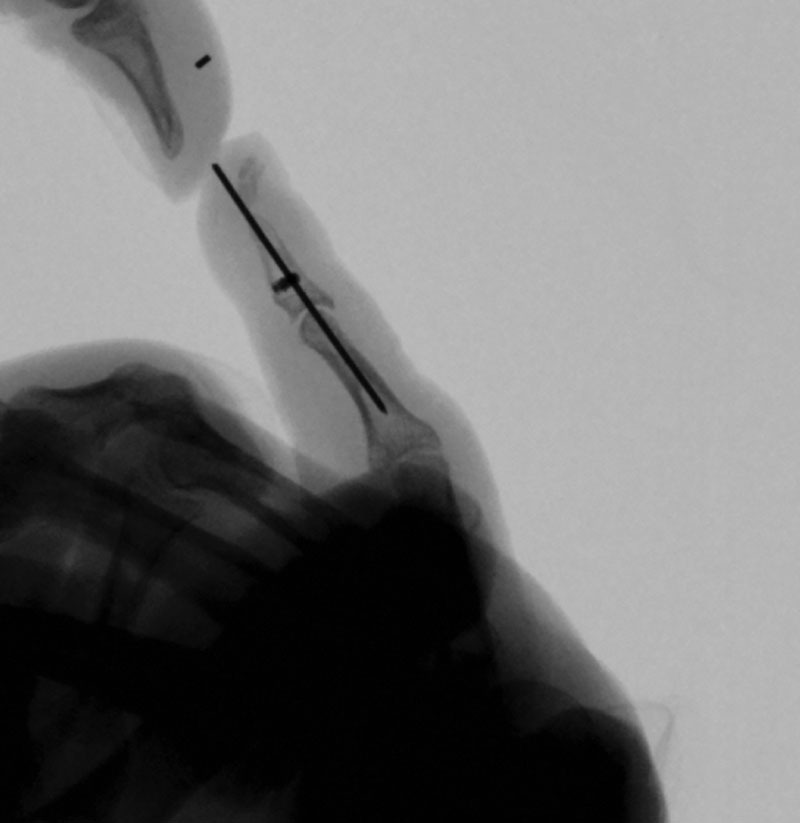
DIP joint pinning and placement of suture anchors.

The distal phalanx was accessed through a mid-axial incision. Discontinuity of pulp tissue from the distal phalanx created an easy plane between pulp tissue and periosteum for exposure. DIP capsulotomy, volar plate release, and pinning with a buried Kirschner wire were performed to correct the joint contracture. Two micro-bone anchors (Microfix, DePuy-Mitek, Raynham, MA) were inserted at the junction of the proximal and middle third of P3 (Fig. 3). Pulp tissue was draped in anatomic position, and the anchor sutures were provisionally placed in the pulp to ensure alignment. 0.5 mL of fibrin glue (Tisseel Fibrin, Baxter Healthcare Corporation, Deerfield, IL) and 0.5 mL of sterile talcum powder were applied in the plane between the periosteal and soft tissue layers, and the anchor sutures were subsequently tied down, taking care to avoid strangulation of soft tissue.

The patient healed uneventfully. At 1 month, the pin was removed, restrictions lifted, and work hardening initiated in physical therapy. At 2.5 months, patient reported improved stability and grip function of the small finger. Grip measurement improved from 10 to 60 pounds postoperatively. Semmes testing improved from 3.84 to 2.83. He demonstrated no instability or pain on sheer testing. Although he did develop a mild recurrence of his DIP flexion contracture (25 degrees on final evaluation, compared to 45 preoperatively), he had no further protuberant P3 bone deformity in the mid-volar pulp and he was able to flex the digit completely into the palm. Active range of motion (extension/flexion) improved at all joints: 0/5 to –25/50 at the DIP, –20/30 to –30/80 at the proximal interphalangeal (PIP) joint, and –10/40 to 0/75 at the metacarpophalangeal (MCP) joint.

**Fig. 4.**
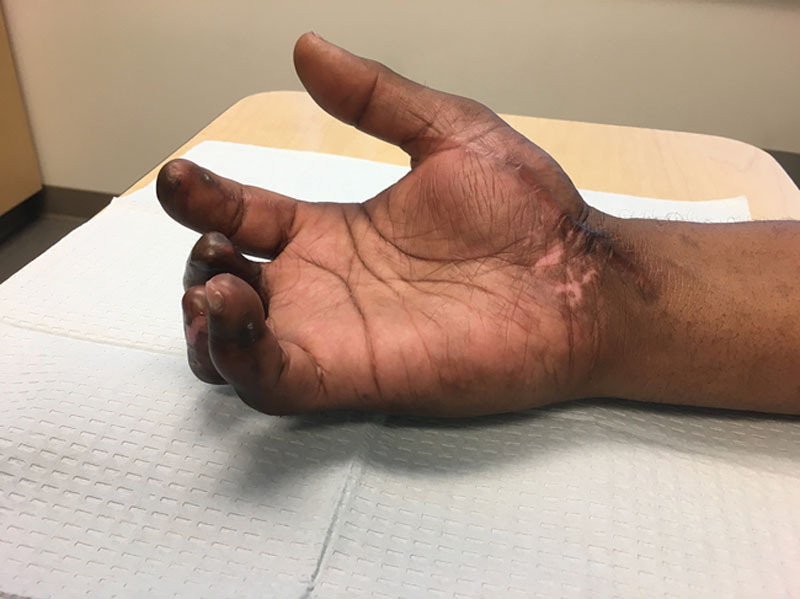
Six months postoperatively.

Although he sustained permanent disability of the hand due to the severity of this initial injury, he reported substantial quality of life improvements with small finger pulp resuspension and postoperative therapy. QuickDASH scores improved from 91 to 59. He resumed hobbies, including drawing and cutting hair, and started a new clerical job without complaints. At 1 year after surgery, his examination remained stable and he continued to report high satisfaction with his outcome (Fig. 4).

## CONCLUSIONS

Digital pulp degloving injuries occur with varying levels of severity and can cause significant disability. In cases of closed injury with intact perfusion, successful resuspension to bone improves function while maintaining native tissue. To our knowledge, García-López et al have reported the only surgical intervention for a degloving injury of the finger pulp tissue, a case study in which thumb pulp was secured to the distal phalanx with absorbable sutures.^3^ The patient returned to work as a machinist 8 weeks after surgery and was asymptomatic at 6 months postoperatively.

Our technique offers several advantages. Fixation of the avulsed soft tissue with bone anchor is an effective, fast, and relatively simple way to secure stability against shearing forces. Dissection is minimal. Fibrin glue and talcum powder were applied to promote scarring and adherence. Fibrin glue has been shown to increase adherence and promote take of skin grafts. It may have antibacterial properties as well, as bacteria have been shown to grow more slowly in fibrin glue than in physiological clots.^4^ Potential complications with this technique include hardware failure, migration of the bone anchor, iatrogenic fracture, infection, and painful scarring, although none were encountered in this case.

Following the success of this case, a patient presented with a 30-year history of internal degloving injury to the left heel, resulting in pain and instability during ambulation. He underwent 2 reconstructive flap surgeries with repeated infection and persistent symptoms. Three mini-suture anchors, fibrin glue, and talcum powder were used to resuspend the glabrous tissue to the calcaneus. The patient did require 1 additional formal washout due to infection (anchors were salvaged), but ultimately healed well with heel stability and painless ambulation in 15 weeks.

We encourage consideration of this technique for internal degloving injuries on the distal extremities.

## ACKNOWLEDGMENT

Institutional Review Board approval was not required as per institutional guidelines.
